# Odontophobia Across the Lifespan: Clinical Perspectives, Vulnerable Populations, and Inclusive Strategies for Dental Anxiety Management

**DOI:** 10.3390/jcm14165766

**Published:** 2025-08-14

**Authors:** Antonio Fallea, Simona L’Episcopo, Aurora Palmigiano, Giuseppe Lanza, Raffaele Ferri

**Affiliations:** 1 Oasi Research Institute-IRCCS, Via Conte Ruggero n. 73, 94018 Troina, Italy; afallea@oasi.en.it (A.F.); slepiscopo@oasi.en.it (S.L.); rferri@oasi.en.it (R.F.); 2 Department of Surgery and Medical-Surgical Specialties, University of Catania, Via Santa Sofia n. 78, 95123 Catania, Italy; aurora.palmigiano@unict.it

**Keywords:** odontophobia, dental anxiety, vulnerable populations, behavioral dentistry, inclusive oral healthcare

## Abstract

Odontophobia, defined as the intense and persistent fear of dentists or dental care, is a widely underestimated, yet clinically significant, barrier to oral health. It affects individuals across all age groups, from children to the elderly, and is particularly prevalent among those with intellectual or developmental disabilities. Odontophobia is a multifactorial condition influenced by psychological, sensory, cognitive, and sociocultural factors. Left unaddressed, it contributes to poor oral health outcomes, avoidant behavior, and broader health disparities. This perspective paper explores the clinical manifestations and principles of management of odontophobia across populations and different age groups, highlighting the limitations of pharmacological sedation, especially when used in isolation. Instead, evidence supports the use of cognitive behavioral strategies, desensitization protocols, sensory-adaptive environments, and communication-based approaches, such as the “tell-show-do” method. Innovative technologies, including virtual reality, offer additional promise. This paper also addresses critical gaps in the research, the paucity of tailored interventions for vulnerable groups, and both ethical and legal complexities surrounding consent, autonomy, and equitable access. Ultimately, managing odontophobia requires a shift toward “person-centered” and “trauma-informed” dental care, supported by interdisciplinary collaboration, inclusive infrastructure, and policy-level commitment to reduce fear-based disparities in oral health.

## 1. Introduction

Odontophobia is a persistent and disproportionate fear of dental procedures and related settings and is a complex and underestimated phenomenon that can significantly hinder access to dental care and negatively impact individuals’ quality of life [1,2,3]. Its prevalence, cutting across age, gender, and socioeconomic strata, renders it a widespread barrier to oral health and a public health concern in both developed and developing settings [4]. This condition affects both neurotypical individuals and those with different types of disabilities, two populations who, while sharing the same fundamental fear, face profoundly different challenges in coping strategies and accessing appropriate care pathways [5].

In neurotypical individuals, odontophobia frequently emerges as a learned response to previous traumatic dental experiences or from the anticipation of pain based on observation or suggestion. Cultural narratives, family anecdotes, and exaggerated or negative portrayals of dentistry in media further reinforce avoidant behavior [6]. The clinical presentation may range from mild anticipatory discomfort to intense anxiety attacks that impair the patient’s ability to undergo even basic dental procedures. These symptoms often lead to missed appointments, poor adherence to follow-up, and progressive deterioration of oral health. Left untreated, odontophobia may become chronic, affecting both mental and physical health [6].

In this context, the terms “dental anxiety” and “odontophobia” are often mistakenly used interchangeably, although they describe distinct psychological responses. Dental anxiety is typically situational and arises in response to an undefined or anticipated dental stimulus, especially in individuals who may not have had direct negative experiences [7]. Odontophobia, by contrast, represents a specific, intense, and persistent fear directed toward clearly identifiable objects or procedures in dental settings. It is often accompanied by active avoidance behavior, to the extent that individuals seek care only when confronted with severe symptoms that can no longer be ignored, such as pain, infection, or dental emergencies resulting from long-term neglect [8,9].

Based on this consideration, this present perspective study addresses the following question: how can clinical, behavioral, and systemic strategies be optimized to manage odontophobia in vulnerable populations across the lifespan, particularly those with disabilities, neurodivergence, or age-related impairments? By offering a conceptual synthesis of existing evidence, clinical observations, and population-specific challenges in the management of odontophobia, we aim to highlight neglected dimensions of care, propose inclusive clinical strategies, and identify future research priorities.

To this end, we consulted the literature targeting peer-reviewed publications relevant to odontophobia in vulnerable populations. We primarily used PubMed and applied the following search terms from database inception: odontophobia, dental anxiety, intellectual disability, cognitive behavioral therapy, virtual reality dentistry, pediatric dental fear, and inclusive oral health. The reference lists of key systematic reviews and meta-analyses were also hand-searched to identify additional relevant studies. Inclusion focused on peer-reviewed original articles, systematic reviews, and meta-analyses, as well as studies addressing behavioral, psychological, environmental, or technological interventions and populations including children, adults, the elderly, and individuals with disabilities. Nonetheless, a formal systematic methodology was beyond the target of this “Perspective” article.

## 2. Odontophobia in Individuals with Disabilities

Among individuals with disabilities, whether cognitive, sensory, motor, or mixed, the situation becomes more complex. In this population, fear is not only linked to trauma or anticipated pain but is aggravated by communication difficulties, sensory hypersensitivity, and clinical environments that are often unequipped to meet their needs. Limited access to specialized dental care, a shortage of trained professionals, and the absence of dedicated protocols further exacerbate the issue, turning odontophobia into a real form of healthcare exclusion [10].

In subjects with intellectual disability, dental fear is particularly prevalent due to the high rate of significant functional impairments and co-occurring psychiatric conditions, which make dental procedures extremely challenging. Moreover, several studies indicate that individuals with disabilities are more susceptible to developing dental anxiety due to difficulty in understanding the procedures, a lack of environmental control, and, in many cases, the use of physical restraint, which is experienced as traumatic [11]. Paradoxically, those requiring the most dental care are often the most excluded from it.

Sensory processing difficulties, maladaptive behaviors, and dental anxiety complicate both routine care and more specialized interventions. Specifically, individuals with intellectual and developmental disabilities (IDD) are disproportionately vulnerable to poor oral health compared to typically developing peers due to numerous risk factors contributing to their complex needs [12,13,14]. People with IDD commonly present with motor, perceptual, linguistic, sensory, cognitive, and behavioral impairments that can create or worsen challenges in performing daily oral hygiene [15,16]. Caregivers frequently report difficulties even in brushing the teeth of these patients, and poorer oral hygiene practices have been associated with plaque buildup, gingival inflammation, and an increased risk of dental caries and related complications [12,13,14,17].

As such, identifying odontophobia in individuals with IDD, particularly those with severe intellectual and/or motor impairments, presents unique diagnostic challenges. Standardized diagnostic tools, such as the Modified Dental Anxiety Scale [7], the Dental Fear Survey [18], and the Children’s Fear Survey Schedule–Dental Subscale [19], have been widely used in general populations, although they may not be directly applicable or valid in patients with limited verbal communication or cognitive impairment [1,2]. In such cases, behavioral observations, caregiver reports, and physiological indicators (e.g., heart rate, sweating, agitation) are often used as a proxy for fear assessment [20,21].

Key diagnostic features of odontophobia in this population include consistent avoidance of dental settings, heightened distress during oral hygiene routines, and significant emotional or behavioral dysregulation in anticipation of dental care. Criteria should also consider the persistence and intensity of fear, its impact on oral health maintenance, and the need for sedation or restraint to complete even non-invasive procedures [22]. More adapted scales and observational frameworks are being developed to better capture fear responses in individuals with IDD, which underscores the urgent need for validated, inclusive diagnostic criteria tailored to this vulnerable group [9,23].

## 3. Traditional Management of Odontophobia

Despite the clinical and public health challenges posed by odontophobia, several effective management strategies have been identified across behavioral, environmental, educational, and pharmacological domains. Historically, odontophobia was often addressed primarily through pharmacologic means, such as oral or intravenous sedation or, in selected cases, general anesthesia. However, such approaches, while sometimes necessary, do not address the underlying psychological causes of fear and may reinforce avoidance behavior, especially if used in isolation. A more comprehensive and sustainable approach emphasizes non-pharmacologic techniques aimed at empowering patients, modifying their perceptions of dentistry, and fostering trustful therapeutic relationships. Indeed, behavioral therapy has been shown to outperform inhalation sedation [24].

A cornerstone of modern behavioral management is graduated exposure, wherein fearful patients are gradually introduced to dental stimuli in a controlled and non-threatening manner, allowing for systematic desensitization. This is often combined with cognitive behavioral therapy (CBT) techniques aimed at reframing catastrophic beliefs and improving self-efficacy. In this context, a systematic review supports the efficacy of behavioral interventions (including CBT, desensitization, relaxation techniques, and hypnosis) in reducing odontophobia in adult patients [25].

## 4. Virtual Reality in the Management of Odontophobia

Beyond behavioral approaches, technological tools are gaining prominence in addressing dental fear. Virtual reality (VR) is emerging as a promising non-pharmacological tool in the management of odontophobia, particularly in pediatric and neurodivergent populations. Notably, Barros Padilha et al. [26] identified VR as an innovative intervention capable of alleviating both odontophobia and procedural pain in children. In their systematic review, nine out of eleven included studies reported a statistically significant reduction in dental anxiety scores when VR was used as a distraction method during treatment. Specific outcomes included a mean reduction of 1.9 points on the Facial Image Scale and an over 30% decrease in self-reported pain scores in pediatric patients [26]. In particular, it has been noted that the immersive, interactive nature of VR enables patients to be transported into calming or entertaining virtual environments, shifting their attention from dental procedures and thus reducing perceived distress. Notably, this form of sensory distraction may not only enhance patient cooperation but also improve the overall treatment experience.

The clinical relevance of such approaches is underscored by findings from Wu et al. [27], who reported that approximately one in four children and adolescents experience dental fear and anxiety, often resulting in avoidant behaviors that can persist into adulthood. In more detail, the authors described a randomized trial protocol aimed at assessing the impact of a VR-based game on reducing behavioral distress and anxiety in children during dental treatments, suggesting a growing trend toward standardized implementation and evaluation of immersive technology. These findings underscore the potential of VR not only as a distraction technique but also as a structured therapeutic adjunct that can enhance cooperation, reduce reliance on sedation, and improve patient satisfaction, particularly in populations with heightened sensory or emotional vulnerability [27]. This fear can lead to treatment delays, deterioration in oral health, and greater reliance on invasive procedures. Clinically, such patient uncooperativeness may disrupt workflow, prolong appointments, and, in some cases, necessitate the premature termination of procedures or the use of pharmacologic sedation.

VR also represents a valuable strategy for managing behavioral challenges, especially among children with special healthcare needs. By offering a multimodal, engaging experience, it facilitates emotional regulation and attentional redirection. Mehrotra et al. [28] compared the effectiveness of audio distraction versus VR in children with mild intellectual disabilities and found both modalities to be similarly effective in reducing anxiety and supporting behavioral compliance during treatment. In particular, the VR group showed a greater decrease in anxiety scores as measured by the Venham Picture Test, with average scores decreasing from 4.8 to 1.6 compared to 4.9 to 2.9 in the audio group [28]. The study concluded that VR was more effective in facilitating behavioral cooperation and minimizing distress during dental procedures, thus promoting the incorporation of VR-based protocols as a part of an inclusive and patient-centered dental care approach.

Finally, a recent review by Pisano et al. [29] examined the combined use of audiovisual (AV) and VR tools for pediatric patients with special healthcare needs, proposing a multi-session model called “UNISA-Virtual Stepwise Distraction”. This approach progressively combines traditional and AV–VR techniques to familiarize children with the dental setting and manage anxiety and behavior. The model includes pre-visit familiarization sessions using “tell-show-do” and visual aids; the gradual introduction of AV content (2D videos, tablets, AV glasses) to promote distraction; the controlled use of immersive VR, such as full-coverage headsets showing relaxing or playful environments in later sessions, if tolerated; and behavioral desensitization sessions, which combine VR with “tell-show-do” and positive reinforcement to increase cooperation. Overall, this stepwise method has shown positive feedback from caregivers and practitioners, reducing stress and improving behavior and treatment adherence [29]. Visual impairments, photosensitive epilepsy, severe sensory sensitivities, or profound cognitive behavioral-related issues may be possible limitations; similarly, untrained operators may face difficulties integrating VR into clinical routines. Nevertheless, for patients with mild-to-moderate cognitive or sensory impairments, VR within this stepwise model significantly reduces anxiety (up to 50% on certain scales), increases treatment tolerance, and decreases the need for sedation. However, in severe disabilities or non-verbal cases, VR can be overstimulating and should be replaced with simpler AV distractions or sensory-adapted dental environments [29].

## 5. Odontophobia in Children: Early Onset of a Lifelong Barrier

In children, dental fear may appear early and escalate without proper intervention. Pediatric odontophobia, often underestimated in clinical settings, often represents the initial stage of dental fear that can evolve into chronic avoidance behaviors across the lifespan. Studies estimate that 9–20% of children experience clinically significant levels of dental fear or anxiety, which can interfere with routine dental care and lead to the deterioration of one’s oral health status over time [18,27]. In this population, fear is shaped by a unique set of cognitive, emotional, and environmental factors. These include a limited understanding of dental procedures, lower pain tolerance, and previous negative experiences (e.g., painful extractions or restraint), as well as modeling parental anxiety [5,30].

## 6. Odontophobia in Adulthood: Chronicity, Comorbidity, Complexity

In adults, odontophobia presents as a deeply ingrained, often debilitating condition with significant impacts on oral and general health. It is frequently the culmination of a lifelong trajectory of avoidance, shaped by unresolved childhood dental trauma, social embarrassment, and comorbid psychopathologies, such as generalized anxiety disorder, panic attacks, depression, or post-traumatic stress disorder [2,3,9].

Despite its prevalence and burden, odontophobia in adults remains underdiagnosed and undertreated. However, a range of effective psychological treatments exist, including CBT, exposure-based interventions, relaxation training, hypnosis, and systematic desensitization. These therapies target maladaptive beliefs, reduce physiological arousal, and gradually reintroduce patients to feared stimuli in a controlled and supportive manner. Nevertheless, access to such treatments is limited by stigma, cost, lack of specialized providers, and the insufficient integration of psychological services into dental settings [3].

## 7. Odontophobia in the Elderly: A Neglected Problem, a Neglected Population

While odontophobia is widely recognized in pediatric and general adult populations, its manifestation in older adults remains underexplored. Nevertheless, the aging population is uniquely vulnerable to the consequences of dental fear, with clinical, psychological, and systemic factors compounding both the expression of anxiety and the challenges of accessing care. A population-based study of adults aged 50–89 years found that 8.4% exhibited clinically significant odontophobia, which correlated with delayed or missed dental visits (clear evidence of avoidance behavior in older adults) [31].

In older adults, dental fear may be linked not only to previous traumatic dental experiences, often in eras when pain control was less effective, but also to functional decline, chronic illness, cognitive impairment, and polypharmacy, all of which can amplify perceived vulnerability in clinical settings. Sensory changes, such as diminished vision or hearing, as well as mobility limitations and cognitive slowing, may further increase anticipatory anxiety and perceived helplessness, especially in environments not adapted to their needs [32]. Odontophobia in this age group is also exacerbated by loss of autonomy and increased dependency on caregivers. For instance, for institutionalized elderly individuals, dental visits may be scheduled by others, limiting a sense of control. In those with neurocognitive disorders (e.g., Alzheimer’s disease or other dementia), even routine procedures can provoke heightened fear or behavioral resistance due to impaired understanding or disorientation, further complicating care. A systematic review focusing on elderly individuals with dementia reported a high prevalence of gingival bleeding, periodontitis, mucosal lesions, and xerostomia, which are all oral conditions that can trigger fear and complicate care [33].

Despite these challenges, few studies have addressed tailored interventions for dental fear in the elderly. Adapted behavioral approaches, including simplified communication, longer appointment times, the use of empathy-focused strategies, and caregiver-assisted desensitization, may be effective, although they have been rarely implemented systematically [34,35]. Likewise, there is a lack of validated tools for assessing dental anxiety in this population, particularly in those with cognitive impairment or sensory loss.

## 8. Main Barriers to Clinical and Research Implementation

Despite promising strategies, several barriers still limit their practical application. Despite the growing recognition of odontophobia as a critical barrier to oral healthcare, the translation of research findings into widespread clinical practice remains limited. Several structural, methodological, and systemic barriers hinder both the development of high-quality evidence and its integration into routine dental care. A qualitative study with private-practice dentists found that while clinicians recognize the value of CBT-based tools, concerns about time, staff capacity, and lack of observable implementation models strongly discourage the adoption of behavioral treatments in everyday practice [36].

From a research standpoint, one major limitation is the lack of standardized and validated diagnostic tools to differentiate between dental anxiety and odontophobia across diverse populations. Existing scales often conflate general anxiety with specific dental fear, whereas only a few are adapted for use in individuals with IDD or children with neurodevelopmental disorders. This limits comparability across studies and the ability to assess treatment effectiveness in real-world settings.

Additionally, despite the diversity of populations affected (i.e., children, adults, the elderly, and individuals with and without disabilities), odontophobia presents recurring cross- and age-population challenges. The main obstacles include the following:

➢ Barriers to access: Sensory disturbances, communication difficulties, and provider bias often lead to delayed or denied care in vulnerable groups;
➢ Over-reliance on sedation: Across populations, sedation is frequently used as a default strategy in the place of behavioral or environmental interventions, limiting long-term desensitization and autonomy;
➢ Lack of standardized assessment: Diagnostic inconsistencies and the absence of adapted tools hinder the identification and monitoring of odontophobia, particularly in patients with IDD or cognitive impairment;
➢ Training gaps: Many dental professionals report limited training in behavioral techniques, inclusive communication, or trauma-informed care, contributing to discomfort or avoidance in treating phobic patients;
➢ Policy and structural limitations: Inclusive care models remain underfunded and poorly disseminated, with little support for multidisciplinary collaboration.

Implementation is further complicated by insufficient behavioral health training in dental education programs in this population. Many dentists report limited confidence or experience in managing highly anxious or phobic patients without resorting to sedation [37]. Integrating behavioral techniques into standard curricula and continuing professional development is essential yet currently remains inconsistent across institutions.

Economic and organizational constraints also represent a major obstacle to adoption. Interventions such as VR equipment, desensitization protocols, or sensory-adapted settings require investments of time, staff training, and resources that may be infeasible in busy general practice or low-resource settings [38]. Additionally, behavioral management strategies are often poorly reimbursed, if at all, by public and private insurance schemes, which disincentivizes their use in favor of faster (e.g., pharmacological) approaches.

Regarding research perspectives, some strengths of the retrieved bibliography include the inclusion of some systematic reviews and meta-analyses (which provide a high level of evidence), the emphasis on emerging modalities like sensory-adaptive environments and VR (also supported by recent controlled studies), and the coverage of diverse populations, including neurodivergent and elderly individuals (who are often under-represented in earlier studies). Nevertheless, weaknesses and limitations are also evident: (i) many studies, especially those involving vulnerable groups (e.g., people with IDD), are small-scale, observational, or case-based, limiting generalizability; (ii) there is a lack of standardized outcome measures across studies (e.g., varying dental anxiety scales), which complicates comparison; (iii) there are few longitudinal studies assessing the durability of interventions or behavioral changes over time; and (iv) there is an under-representation of low- and middle-income countries.

In conclusion, while evidence for managing odontophobia is growing, the inference and overall assessment remain heterogeneous, particularly in terms of methodological rigor and population diversity. The strongest evidence supports CBT, desensitization, and a sensory-adaptive dental environment in children and individuals with IDD, whereas the evidence for older adults and integrated interdisciplinary care is still limited. Future research should prioritize the development of adapted anxiety assessment tools, large-scale trials of behavioral and sensory-adaptive interventions, cost-effectiveness analyses, and dissemination studies to scale up inclusive care models. Policy initiatives must promote training, infrastructure, and equitable access to trauma-informed oral healthcare.

## 9. Legal and Ethical Aspects

Equitable access to care is a key ethical issue. Patients with high levels of dental fear or behavioral challenges are sometimes denied care due to presumed unmanageability. However, failing to provide reasonable accommodations, such as modified environments, communication aids, or extended appointment times, can be construed as discriminatory under disability rights legislation. The duty to ensure inclusivity in dental care is not only ethical but legally grounded in frameworks such as the United Nations Convention on the Rights of Persons with Disabilities [39]. Nonetheless, addressing these barriers requires not only investment in high-quality clinical research but also policy changes that prioritize access to behavioral dentistry, equity in dental education, and reimbursement structures supporting time-intensive and person-centered care.

## 10. Conclusions

Odontophobia is a pervasive and underestimated condition that significantly hinders access to oral healthcare and affects individuals across age groups and cognitive profiles, posing special challenges for vulnerable populations, including those with disabilities and the elderly. Despite increasing recognition of its impact, research and clinical strategies remain fragmented and are often focused narrowly on sedation or pediatric care. Training dental professionals in these approaches, alongside clearer ethical and legal frameworks for managing consent and cooperation, is essential. Technological tools such as VR also show promise and warrant further investigation. Table 1 summarizes key gaps in knowledge and clinical practice, highlights unmet needs, and guides future research directions, whereas Table 2 outlines general recommendations for managing odontophobia across distinct patient populations, based on the literature reviewed in this perspective study.

In addition to its clinical relevance, odontophobia presents an opportunity for innovation in dental care delivery. Policies should prioritize the integration of behavioral strategies into standard dental practice, provide funding for inclusive infrastructure (e.g., sensory-adapted environments), and ensure equitable access to specialized care for neurodivergent, disabled, and elderly individuals. Building clinical pathways that reflect trauma-informed, interdisciplinary, and patient-centered values will be essential to overcoming fear-based disparities in oral health outcomes and reaching supportive policy-level changes.

## Figures and Tables

**Table 1:** Gaps in knowledge and research perspectives on odontophobia.

Knowledge Gap	Clinical Opportunities and Research Perspectives
Lack of standardized diagnostic criteria and tools for odontophobia	Develop validated, age-appropriate, and neurodiversity-sensitive diagnostic instruments
Under-representation of vulnerable populations (e.g., the elderly, individuals with intellectual and developmental disabilities)	Design targeted studies to evaluate fear profiles and effective interventions across diverse and underserved groups
Limited longitudinal data on the progression and impact of odontophobia	Conduct long-term cohort studies to assess outcomes, trajectories, and treatment adherence
Over-reliance on pharmacologic sedation without behavioral integration	Investigate combined approaches integrating behavioral therapy and technology-based distractions (e.g., virtual reality)
Scarcity of randomized controlled trials on non-pharmacological treatments	Support multicenter randomized controlled trials evaluating cognitive behavioral therapy, desensitization, and sensory-adaptive interventions across populations
Inadequate training of dental professionals in behavioral techniques	Incorporate odontophobia-specific modules into dental curricula and continuing education programs
Ethical uncertainty in managing non-cooperative patients	Develop ethical guidelines and legal frameworks for consent, restraint, and inclusive care pathways
Limited dissemination of inclusive care models	Promote implementation research on scalable, person-centered, and trauma-informed dental care practices

**Table 2:** Population- and age-specific main recommendations for managing odontophobia.

Population/Age Group	Recommended Interventions
Neurotypical children and adolescents	- Use age-appropriate language and visual aids (e.g., social stories) - Apply “tell-show-do” and positive reinforcement - Use distraction techniques (e.g., virtual reality, music, etc.) - Educate and involve caregivers - Avoid restraint unless absolutely necessary
Individuals with intellectual and developmental disabilities	- Create sensory-adapted environments (e.g., noise control, dim lighting) - Use caregiver-assisted desensitization and visual supports - Apply neurodiversity-sensitive communication - Train staff in inclusive behavioral techniques
Neurotypical adults with odontophobia	- Screen for psychiatric comorbidities (e.g., anxiety, post-traumatic stress disorder) - Offer cognitive behavioral therapy and exposure therapy - Build continuity with familiar dental providers - Reduce reliance on pharmacologic sedation
Older adults/elderly individuals	- Simplify communication and extend appointment durations - Use empathy-based approaches and continuity of care - Involve caregivers and observe behavioral cues in case of cognitive decline - Avoid overstimulation and rushed procedures
Institutionalized or medically complex patients	- Provide mobile/home-based services when feasible - Collaborate with interdisciplinary teams - Ensure adapted informed consent procedures - Document accommodations and behavioral responses thoroughly

## Abbreviations

The following abbreviations are used in this manuscript:

AV	audiovisual
CBT	cognitive behavioral therapy
IDD	intellectual and developmental disabilities
VR	virtual reality
